# Can Primary Knee Tuberculosis Simulate the Clinical and Radiological Profile of Pigmented Villonodular Synovitis?

**DOI:** 10.7759/cureus.70891

**Published:** 2024-10-05

**Authors:** Moulay Rchid Ismail, Samir Ben Salah, Adnane Lachkar, Najib Abdeljaouad, Hicham Yacoubi

**Affiliations:** 1 Department of Traumatology and Orthopedics B, University Hospital Mohammed VI, Oujda, MAR; 2 Department of Orthopedic Trauma, Faculty of Medicine and Pharmacy, University Hospital Mohammed VI, Oujda, MAR; 3 Department of Orthopedic Trauma, Faculty of Medicine and Pharmacy, University Hospital Mohammed VI, Mohammed First University of Oujda, Oujda, MAR; 4 Department of Traumatology and Orthopedics, Faculty of Medicine and Pharmacy, University Hospital Mohammed VI, Mohammed First University of Oujda, Oujda, MAR

**Keywords:** knee, pigmented, primary, tuberculosis, villonodular synovitis

## Abstract

When faced with clinical and radiological findings suggestive of villonodular synovitis, tuberculosis is not often considered a differential diagnosis, especially when the patient is not a known tuberculosis carrier. In this paper, we present an exceptional case of a patient who had a tumefaction (measuring 17 cm in length) in the anterointernal region of her left knee, with a clinical and radiological picture in favor of villonodular synovitis. However, after tumor resection, the anatomopathological study of the surgical specimen came back in favor of a tuberculous lesion. This exceptional case shows that tuberculosis should be retained as a diagnostic possibility in the presence of clinical and radiological findings in favor of villonodular synovitis, even if the patient is not known to have a tuberculous lesion elsewhere.

## Introduction

Villonodular synovitis (VNS) is a disease of young adults (30-40 years) that can also affect children. It is more common in women, most commonly affects the knee (between 63% and 75%), and is monoarticular in most cases [1]. It is a benign tumor, the pathophysiology of which is not entirely clear; it can be a tumor or inflammatory in origin [2].

## Case presentation

In this paper, we present our experience with a patient who was treated in our Traumatology and Orthopaedics Department. The patient was 40 years old, had a pathological history of cervical cancer (two years ago), was treated with radiotherapy and chemotherapy combined with brachytherapy, and had a favorable outcome.

She consulted us after the appearance of a localized swelling in the anteroinferior region of her left knee, which was first discovered by auto palpation six years ago. The swelling had progressively increased in size, accompanied by unquantified weight loss and intermittent pain relieved by the usual analgesics, without signs of inflammation.

**Figure 1:**
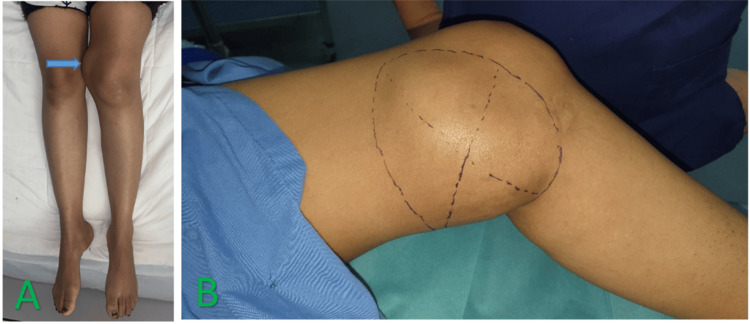
Clinical appearance of the tumor in the prone position (A) showing tumor margins (B)

**Figure 2:**
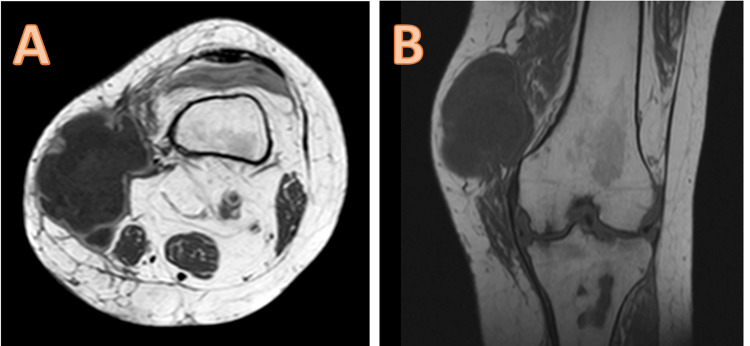
MRI appearance in axial (A) and frontal (B) sections without contrast product injection

**Figure 3:**
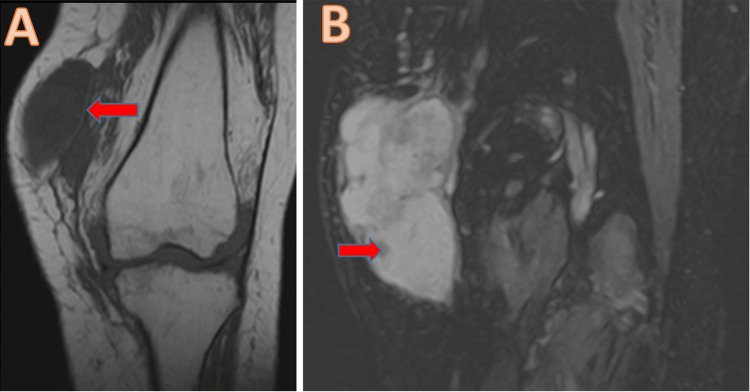
MRI frontal section showing capsule thickening (A) with hemosiderin deposits (B)

The results of the clinical examination showed the presence of a localized swelling in the anteroinferior region of the left knee, measuring 17 cm in length, with a tenacious consistency, painful to palpation, mobile in the superficial plane, and adherent in the deep plane, with a limitation of the joint amplitudes of the knee (Figure 1). It was responsible for functional discomfort when walking due to its volume. For radiological examination, the patient underwent an MRI of the left knee in T2 and T1 sequences, with and without gadolinium injection, showing aggressive villonodular synovitis with subchondral bone lacunae of the medial tibial plateau (Figures 2, 3).

**Figure 4:**
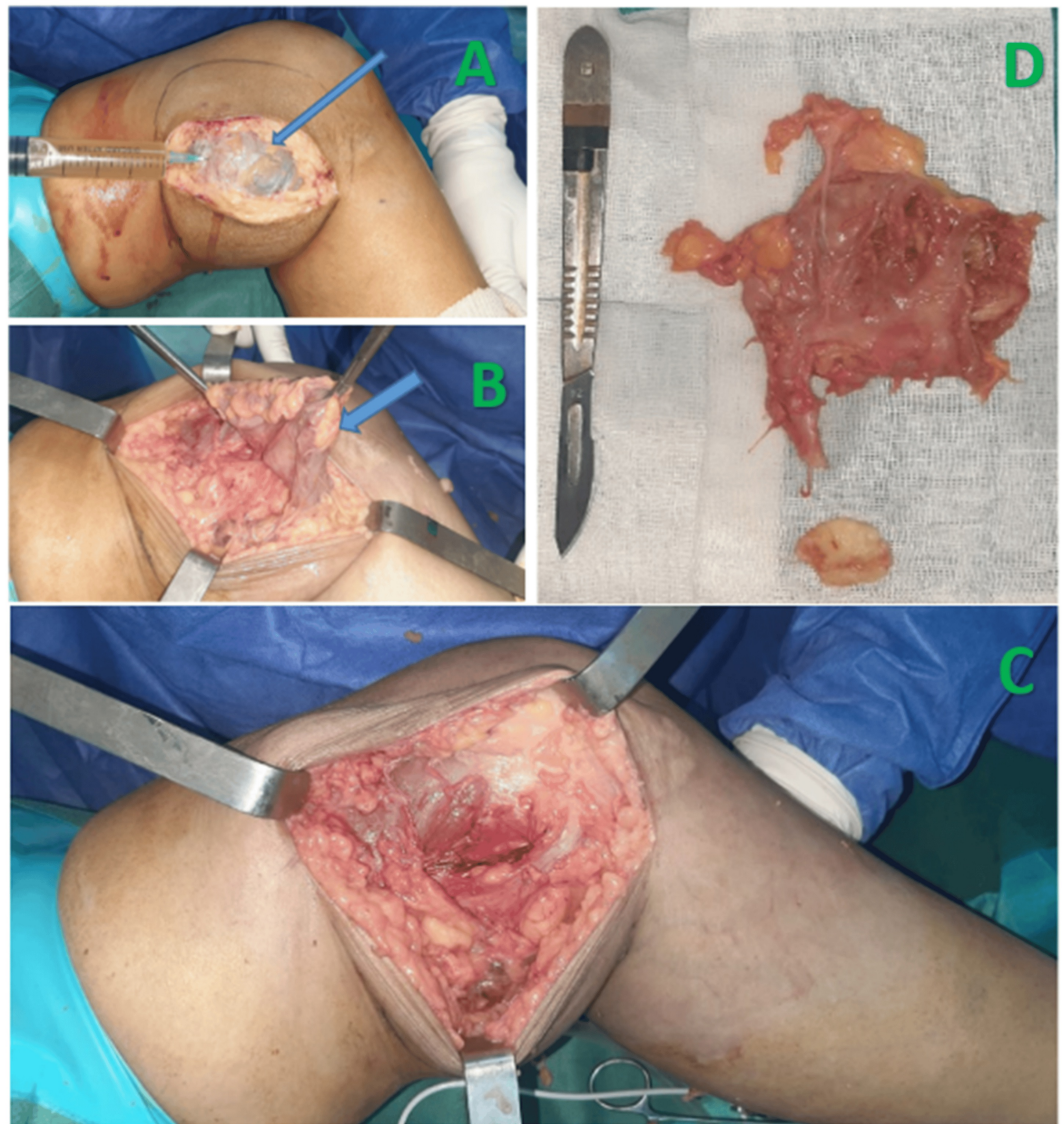
Intraoperative images during tumor resection A and B: Macroscopic appearance of the tumor; C: Appearance after resection; D: Aspect of the surgical specimen

**Figure 5:**
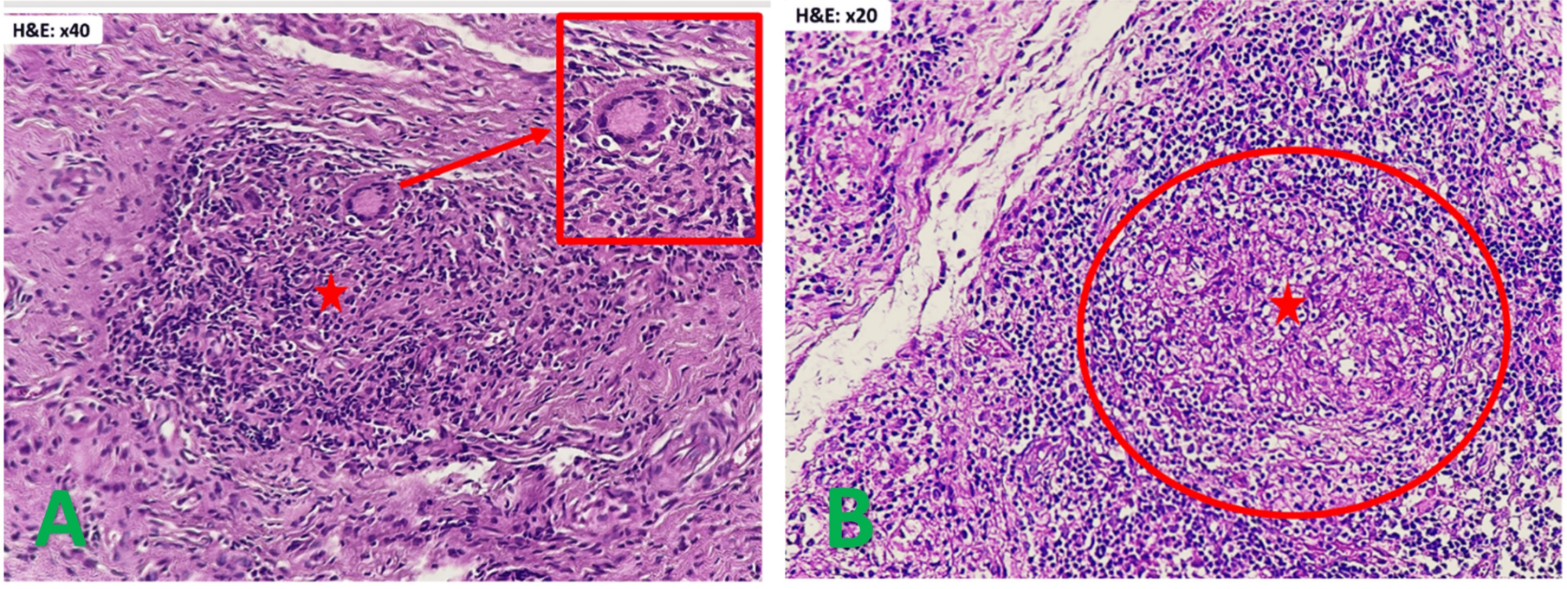
Pathological findings of the surgical specimen A: Epitheliogiganto-cellular granuloma with early caseous necrosis; B: Epitheliogiganto-cellular granuloma

The patient initially underwent a biopsy of the tumor, which ruled out a malignant process. However, the etiology of the swelling could not be determined from the first biopsy. Finally, the patient was taken to the operating theatre for tumor resection, where the tumor was well-circumscribed and encapsulated, filled with non-pigmented, citrine-yellow fluid, with a base of implantation on the medial surface of the knee synovium (Figure 4). Anatomopathological examination revealed granulomatous and epithelioid gigantocellular synovitis with focal caseous necrosis, without anatomopathological evidence of villonodular synovitis (Figure 5). The patient received antibacterial treatment for nine months and was reviewed at two years with a favorable outcome and no signs of recurrence.

## Discussion

Villonodular synovitis is evoked by a slowly evolving picture of painful swelling associated with hemarthrosis. MRI is the test of choice in cases of clinical suspicion of VNS to investigate and further guide the diagnosis; synovial thickening is seen, typically with a hypointense image in T1 and T2 due to the hemosiderin deposits characteristic of VNS [3,4]. However, these clinical and radiological signs are not specific to VNS and may correspond to other diagnoses, such as hemophilic arthropathy, amyloid arthropathy, chronic hemarthrosis, chronic tophaceous gout, chondrocalcinosis, long-standing rheumatoid arthritis, synovial hemangioma, synovial osteochondromatosis, and osteoarthropathy of the nerves [5]. The differential diagnosis of VNS does not include primary tuberculosis, given the suggestive clinical and radiological picture [6,7]. In our case, the terrain (a young woman), the clinical picture (painful swelling of the knee with the presence of effusion), as well as the radiological signs on MRI were very suggestive of VNS, but the anatomopathological study of the surgical specimen after tumor resection confirmed that it was tuberculosis. Given that tuberculosis is not described as a differential diagnosis of VNS, this rare case described in our paper confirms that tuberculosis can take on the clinical picture and signs of VNS [8].

## Conclusions

Our experience with this exceptional case of tuberculosis of the knee, which clinically and radiologically simulated VNS, opens our minds to another differential diagnosis of VNS, which should also be considered even if the patient is not a known carrier of tuberculosis infection, as one of the other diagnoses to be evoked in the face of a clinical and radiological picture in favor of VNS. This work allows us to start a broad search for similar cases to confirm primary tuberculosis as an important differential diagnosis of VNS in a more substantial sample of patients.
